# Intracerebral Transplantation of Autologous Mesenchymal Stem Cells Improves Functional Recovery in a Rat Model of Chronic Ischemic Stroke

**DOI:** 10.1007/s12975-023-01208-7

**Published:** 2023-11-02

**Authors:** Max I. Myers, Kevin J. Hines, Andrew Gray, Gabrielle Spagnuolo, Robert Rosenwasser, Lorraine Iacovitti

**Affiliations:** 1 Department of Neuroscience, Sidney Kimmel Medical College, Thomas Jefferson University, 900 Walnut Street, Suite 462, Philadelphia, PA 19107 USA; 2 The Joseph and Marie Field Cerebrovascular Research Laboratory, Sidney Kimmel Medical College, Thomas Jefferson University, 900 Walnut Street, Suite 462, Philadelphia, PA 19107 USA; 3 Vickie & Jack Farber Institute for Neuroscience, Sidney Kimmel Medical College, Thomas Jefferson University, 900 Walnut Street, Suite 462, Philadelphia, PA 19107 USA; 4 Department of Neurological Surgery, Sidney Kimmel Medical College, Thomas Jefferson University, 900 Walnut Street, Suite 462, Philadelphia, PA 19107 USA

**Keywords:** Middle cerebral artery (MCA), Mesenchymal stem cell (MSC), Allogeneic, Autologous, Stroke

## Abstract

While treatments exist for the acute phase of stroke, there are limited options for patients with chronic infarcts and long-term disability. Allogenic mesenchymal stem cells (alloMSCs) show promise for the treatment of stroke soon after ischemic injury. There is, however, no information on the use of autologous MSCs (autoMSCs), delivered intracerebrally in rats with a chronic infarct. In this study, rats underwent middle cerebral artery occlusion (MCAO) to induce stroke followed by bone marrow aspiration and MSC expansion in a closed bioreactor. Four weeks later, brain MRI was obtained and autoMSCs (1 × 10^6^, 2.5 × 10^6^ or 5 × 10^6^; *n* = 6 each) were stereotactically injected into the peri-infarct and compared to controls (MCAO only; MCAO + PBS; *n* = 6–9). Behavior was assessed using the modified neurological severity score (mNSS). For comparison, an additional cohort of MCAO rats were implanted with 2.5 × 10^6^ alloMSCs generated from a healthy rat. All doses of autoMSCs produced significant improvement (54–70%) in sensorimotor function 60 days later. In contrast, alloMSCs improved only 31.7%, similar to that in PBS controls 30%. Quantum dot–labeled auto/alloMSCs were found exclusively at the implantation site throughout the post-transplantation period with no tumor formation on MRI or Ki67 staining of engrafted MSCs. Small differences in stroke volume and no differences in corpus callosum width were observed after MSC treatment. Stroke-induced glial reactivity in the peri-infarct was long-lasting and unabated by auto/alloMSC transplantation. These studies suggest that intracerebral transplantation of autoMSCs as compared to alloMSCs may be a promising treatment in chronic stroke.

**Supplementary Information:**

The online version contains supplementary material available at 10.1007/s12975-023-01208-7.

## Introduction

Stroke is the second leading cause of death and a major cause of disability worldwide [1] with ischemic stroke accounting for almost 70% of all cerebrovascular events [2]. Throughout the past two decades, the management of ischemic stroke has progressively changed, shifting from an approach limited to secondary prevention to one focusing on early reperfusion strategies. Intravenous (IV) thrombolysis with recombinant tissue-type plasminogen activator (rt-PA), the only drug approved for the treatment of ischemic stroke [3], while efficacious, must be administered in a brief 4.5 h therapeutic window after symptom onset [4–8]. More recently, intra-arterial (IA) mechanical thrombectomy has become the standard of care for acute ischemic stroke caused by large vessel occlusion in the anterior circulation within 24 h from the onset of symptoms [9–11].

While these potentially effective time-dependent treatments exist for the acute phase of stroke, fewer than 10% of stroke patients are eligible for intra-arterial reperfusion procedures [12]. In addition, there are few, if any, options for patients with chronic infarcts and long-term disability. Thus, after the first 6 months of physical and occupational therapy, most patients have reached a plateau in their recovery, with no approved treatments to further ameliorate sensory, motor, or cognitive deficits [13–15].

Over the last decades, nonclinical studies in rodents and primates, and early clinical studies have investigated the therapeutic potential of various stem cell types revealing MSCs as a promising treatment for stroke [14–31]. In all but a few rat studies [32–34] and one human safety trial [15, 28], the role of MSCs has been studied in an acute model of stroke. Although initially designed as a replacement therapy, MSCs are not thought to replace missing neural circuitry, but rather to act as environmental modifiers, altering the inflammatory landscape of the injured brain (increasing the levels of anti-inflammatory cytokines and growth factors and decreasing the levels of pro-inflammatory cytokines) [21, 31].

To date, MSC transplantation has proven safe and effective in nonclinical [35–41] and clinical investigations [14, 15, 27, 28, 42–47]. In nearly all these studies, MSCs were delivered systemically (IV [37, 38, 40, 41] or IA [35]), within days after stroke. However, the vast majority of MSCs were prevented from reaching the brain by the blood brain barrier. In contrast, when cells were directly implanted in the brain, treatment was shown to be highly effective in acute ischemic stroke models [38, 45, 46]. Regardless of route of administration, in nearly all published studies, allogeneic MSCs (alloMSCs) were used, requiring concomitant treatment with harsh immunosuppressant drugs to prevent the rapid immunorejection of transplanted cells [48, 49]. Except for several rodent studies [35, 40, 50], there remains scant information on the potential utility of autologous MSCs (autoMSCs) which lack immunogenicity. The ability of auto- or alloMSCs to improve recovery in chronic stroke models remains largely unknown.

Therefore, in this study, using STEPS guidelines [14, 51] to closely align preclinical with likely clinical procedures, we sought to determine in rats with chronic strokes from middle cerebral artery occlusion (MCAO) whether autoMSCs, as compared to alloMSCs, delivered directly to the peri-infarct region survive long term at the site of transplantation in the absence of immunosuppressant drugs, affect local glial reactivity, and enhance functional recovery.

## Methods

### Animals

Adult male Sprague-Dawley rats were used in accordance with the Institutional Animal Care and Use Committee (IACUC) at the Thomas Jefferson University, who had approved the use of all animals used in this study. All procedures were done in accordance with institutional guidelines. All animal experiments were conducted following ARRIVE guidelines. Rats were housed in the Thomas Jefferson University BLSB-Animal Facility at a light cycle of 12 h light: 12 h dark with *ad libitum* access to food and water. Rats ranged in weight from 275 to 300 g prior to MCAO surgery. Only rats with a significant infarct of the motor and somatosensory cortices and the striatum on MRI and an mNSS of >9 at 1-month post-MCAO were included in the study of chronic stroke in order to mimic major stroke in humans. Rats with smaller strokes, which often spontaneously recover over several months, were excluded. Many rats experienced temporary weight loss after MCAO, but all recovered within a week (Supplementary Table 1). Rats were then randomly assigned to control (or PBS) or study groups and various doses of MSCs, as outlined in STEPS for dose-response effects, were transplanted 4 weeks after MCAO (Day 0) (See Study Design; Supplementary Fig. 1). This timing was recommended for stem cell research in chronic stroke as outlined by STEPS, which states that testing of cell therapy for chronic stroke should first be studied in animal models (≥1-month post-stroke) [14, 51]. Decreased appetite for 24 h was noted after MSC treatment. Adverse effects and a summary of treatment-associated weight loss are provided in the supplementary information (Supplementary Tables 1 and 2). All animals survived post-transplantation of MSCS or PBS treatment. Study design overview.

### Cerebral Ischemia Model

The MCAO model was used to induce major ischemic stroke in rats. Adult male Sprague-Dawley rats were anesthetized via subcutaneous (SubQ) injection of ketamine hydrochloride (100 mg/kg), xylazine (5 mg/kg), and acepromazine (2 mg/kg). A midline incision was made down the neck to expose the right common carotid artery (CCA) and the bifurcation separating the right external and internal carotid arteries (ECA, ICA). Blood flow was restricted by ligating the ECA and proximal CCA with silk sutures and the ICA with a temporary microclip. A silicone rubber–coated nylon filament (Doccol) was inserted into the lumen of the CCA through a small arteriotomy made above the proximal ligation. The ICA clamp was then removed and the nylon filament carefully advanced into the ICA until it obstructed the middle cerebral artery (MCA). After 120 min, the filament was removed and the CCA was ligated, allowing reperfusion of the brain. The muscular layer of the neck was sutured using an absorbable vicryl suture (Ethicon), and the skin was closed using a 5-0 Nylon suture (Ethicon). Finally, animals were administered subcutaneous 20 mL of saline solution to replace blood volume and monitored post-operatively. Buprenorphine (0.05–0.2 mg/kg SQ) was given for pain management as needed, and animals were observed post-operatively. Most pain issues resolved within 24 h, but definitely by 72 h.

### Preparation of MSCs from Rat Bone Marrow

To obtain autoMSCs, bone marrow was aspirated 16 days following MCAO, and 12 days before autoMSC transplantation. Experimental rats were anesthetized through induction of 3–5% isoflurane and maintained on 1–3% isoflurane in 100% oxygen. The right tibia was aseptically dissected through the skin to the medial aspect of the tibia, and a small hole was drilled to access the cancellous bone. A 22-gauge need was inserted into the bone and aspirated using a syringe containing 0.4 mL of 0.2% heparin (Stemcell Technologies) solution in DPBS (Gibco). Bone marrow was aspirated, followed by two consecutive aspirations with syringes containing 0.5 mL DPBS. The needles were removed, the hole was plugged using bone wax, and the incision was closed using a 5-0 Nylon suture (Ethicon). Rats were awake and fully mobile, usually 10 min following surgery, with no indication of pain from this procedure. Whole bone marrow was incubated with 10 mL of Lysis Buffer (Ebioscience) for 5 min, and then 25 mL of DPBS was added to stop the lysis reaction. The bone marrow was then centrifuged at 1500 RPM for 10 min, and cells resuspended in a mixture containing 1% MEM (Gibco), 1% Glutamax (Gibco), 1% Penstrep (Gibco), and 15% Heat Inactivated-Fetal Bovine Serum (HI-FBS) (Gibco) in complete DMEM (Gibco) and plated on a T25 flask to incubate. After 2 days, the cell culture medium was replaced with a mixture of 15% HI-FBS (Gibco) in Prime XV MSC expansion XSFM (Fujifilm). Thereafter, fresh media were replaced every 3 days. For alloMSCs, bone marrow was aspirated from the tibia of healthy (non-MCAO) rats. Periodically, MSC harvests were verified using lineage differentiation kits (ThermoFisher cat#A1007201, A1007101, A1007001) for chondrogenesis, osteogenesis and adipogenesis and test results were recorded in the batch record.

### Sterile Growth Using Bioreactor

Twenty-one days post-MCAO, and 6 days before transplantation, autoMSCs were released from the adherent flask using 0.25% Trypsin-EDTA (Gibco) and resuspended in expansion media. Cells were then loaded onto a Sterile Growth Quantum Bioreactor (Terumo) for rapid expansion. The day prior to cell seeding, the bioreactor was coated with human fibronectin (Corning) for a duration of 24 h. The cells were then loaded onto the bioreactor and allowed to seed for a duration of 48 h. The cells were then fed with a mixture of 15% HI-FBS in Prime XV MSC expansion media, and lactate levels checked daily to monitor growth. On day 28 (day of autoMSC transplantation), TrypLE (Gibco) was loaded into the bioreactor to release expanded cells, followed by centrifugation at 1500 rpm for 10 min to concentrate the cells. Harvested cells were characterized using Flow Cytometry (BD Celesta). Rat autoMSCs were identified as CD11b^−^, CD45^−^, CD29^+^, and CD90^+^ cells. The total cell count was acquired using the Countess II automated cell counter (ThermoFisher).

### Transplantation of MSCs

Harvested auto- and alloMSCs were then diluted in DPBS to reach their final concentration for transplantation. Rats were anesthetized as described above for MCAO and affixed to a robotic stereotactic system (Neurostar GBM) and randomized for treatment. Using same-day MR imaging, the X, Y and Z coordinates for three trajectories were approximated in the peri-infarct region using a rat stereotactic atlas (Paxinos). Next, a midline incision was made, 3 burr holes were drilled in the skull, and cells were deposited at 5 depths for each trajectory, each 1 mm dorsal to the previous injection. Thus, a total of 15 stem cell depositions in 3 trajectories were implanted at the inferior-, mid-, and superior aspect of the infarct border in each of the 3 trajectories to create vertical streams of cells ascending upward from the edge of the infarct. Each deposit consisted of 1 μL of cell suspension (5 μL/trajectory × 3 trajectories for a total volume of 15 μL/brain). The burr holes were patched using bone wax, and the skin was sutured (Ethicon). Finally, animals were given 10 mL of SubQ normal saline solution to help replace blood volume. Buprenorphine (0.05–0.2 mg/kg SQ) was given for pain management as needed, and animals were observed post-operatively. Most pain issues resolved within 24 h. Animals receiving allogenic grafts were immunosuppressed with cyclosporine A (subcutaneous injection of 10 mg/kg in 0.9% sterile saline) daily starting 3 days prior to transplantation and continually until sacrifice to prevent graft rejection.

### Quantum Dot Nanoparticles to Track Cell Fate

In a separate cell-tracking study, cell fate was tracked within the brain in vivo using both autoMSCs and alloMSCs loaded with 655 nm Quantum-Dot Nanoparticles (Q-dots) (ThermoFisher). In these rats, 1 day before transplantation, 1 μL of Q-dots were diluted in 1 mL of 1X borate buffer. This was then added to 4 mL of the 15% HI-FBS in MSC Expansion media mixture for a total of 5 mL. The entire 5 mL was used to replace the media in the flask overnight, to allow endocytosis of Quantum dots into MSCs. After 24 h, cells were released from the flask and resuspended in DPBS for transplantation, as previously described.

### MR Imaging

Rats underwent MR Imaging (MRI) in order to determine the size of the infarct and the location of the peri-infarct region at day -28 and 0, and at various times after cell transplantation (7, 30, and 60 days). T1 Weighted Images (T1WI), T2 Weighted Images (T2WI), and Diffusion Weighted Images (DWI) were acquired on a 1-Tesla MRI scanner (M7™ Compact MRI System, Aspect Imaging). Rats were anesthetized through induction of 3–5% isoflurane and maintained on 1–3% isoflurane in 100% oxygen throughout the MRI procedure. Following the acquisition of MRI images, lesion volume was measured using each slice of the T2WI and summed together to calculate the total volume of infarct. This was done by using the software (Vivoquant, Invicro) to highlight the infarcted area for each 0.8 mm slice of the MRI. Then, we were able to add the area of each slice to calculate the total volume of the infarct.

### Modified Neurological Severity Scores

Concomitantly with MRI analysis, the modified Neurological Severity Scores (mNSSs) was used to determine neurological function in rats on days -28 (1-day post-MCAO), 0 (day of transplantation) 7, 30, and 60 after MSC transplantation. The mNSS is a combination of motor and sensory tests, including circling and walking behavior, wire grip, resistance to lateral push, forelimb flexion and thorax twisting when suspended by the tail, grasping reflex, and spontaneous activity. Points are given for each task the animal is unable to perform on a scale of 0–16 (normal score — 0, maximum deficit score — 16) (Supplementary Table 3). Behavioral assessments were performed by a blind independent observer.

### Postmortem Histology

On day 60, rats were deeply anesthetized as described above for MCAO before being perfused with a 4% solution of paraformaldehyde in PBS. Rat brains were harvested, and cryosectioned. 25 μm thick coronal cryosections of the brain were mounted onto glass slides and stained for Cresyl Violet. Sections stained for cresyl violet were analyzed for differences in width of corpus callosum at bregma −0.26. Using ImageJ (NIH), pixel distance was standardized against a digital micrometer, and width was determined as the distance from the superior to inferior aspect of the CC at the midline. Moreover, sections were analyzed for immunohistological (IHC) staining with antibodies for Glial Fibrillary Acidic Protein (GFAP) (1:500; Agilent #Z033429-2), Ionized Calcium Binding Adaptor Molecule-1 (IBA-1) (1:500; Synaptic Systems #234308), Ki-67 (1:200; ThermoFisher #MA5-14520), and NeuN (1:200, ThermoFisher #PA5-78499). In five randomly selected stained cross sections, five ROIs (40×/section) were chosen that encircled the infarct on the ipsilateral side or was equivalently located on the contralateral side. Likewise, rats that had received MSCs loaded with QDs were similarly analyzed.

### Statistical Analysis

Data are presented as the Standard Deviation (SD) from the mean or mean plus/minus standard error mean (SEM), as specified in the legend. Statistical analysis of raw mNSS scores, percent mNSS scores change, percent infarct volume change, cell counts, and mean fluorescent intensity were performed using ANOVA or the Student’s *T*-test followed by post hoc Tukey test. All studies used statistical power analyses (alpha=0.05, power=0.8), and previous experience with known protocols to establish the minimum number of N needed per condition to achieve statistically relevant results. A *p*-value less than 0.05 is considered significant.

## Results

### Behavioral Assessment and Effects of Intracerebral autoMSC Transplantation

To assess the functional effects of intracerebral autoMSC transplantation in rats with chronic 4-week-old strokes (approximately 6 months in human), we tested various cell doses assessing behavior over a long-time course. The mNSS, which evaluates a battery of motor and sensory tests, was used to assess behavior 1 day after acute stroke (28 days) and on the day of autoMSC transplantation (day 0) and at various intervals thereafter (7, 30 and 60 days) in three cell dose groups (1 × 10^6^, 2.5 × 10^6^, 5 × 10^6^; *n* = 6 per group). In these experiments, each individual rat served as its own control before and after treatment with autoMSCs. In addition, treatment groups were compared to two other control groups: MCAO+PBS (*n* = 9) and MCAO only (*n* = 6) (see study design; Supplementary Figure 1).

**Fig. 1:**
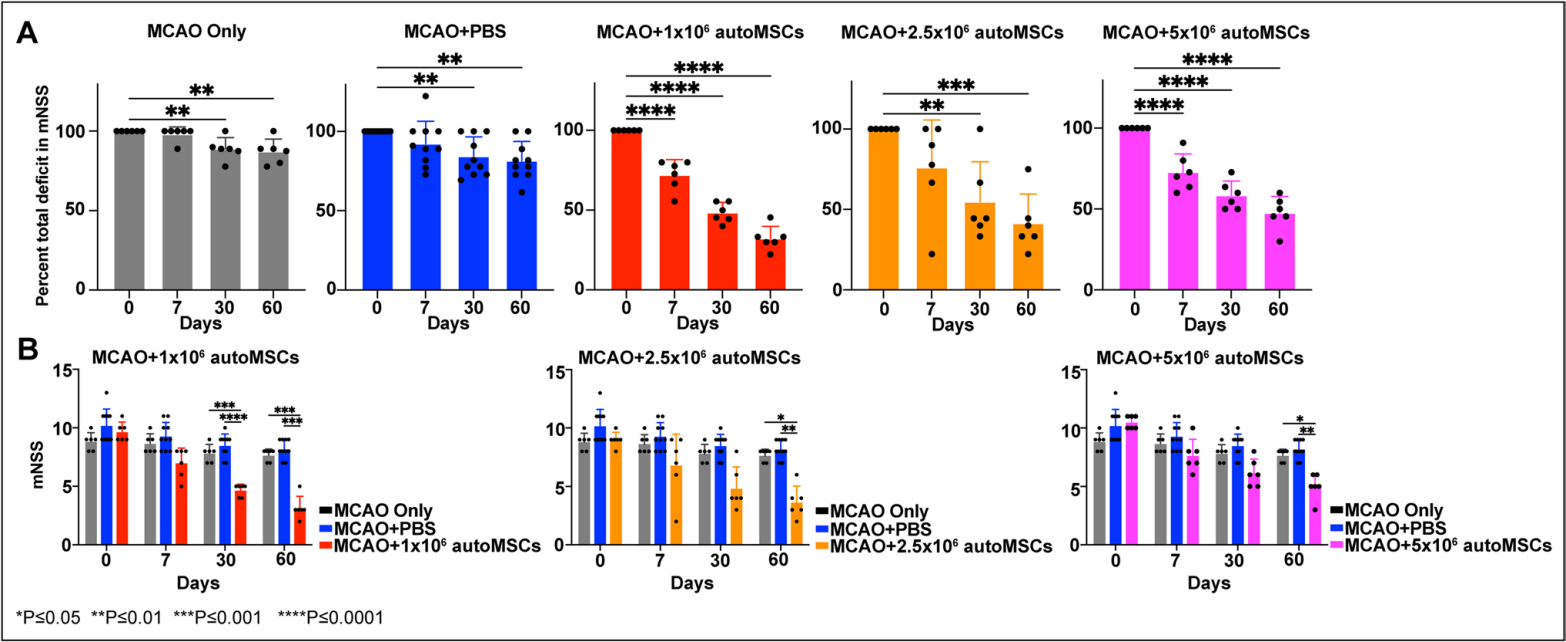
Intracerebrally transplanted autoMSCs produce significant sensorimotor recovery in the chronic stroke brain. (**A**) Behavioral recovery assessed by the Modified Neurological Severity Scale (mNSS) reveals a significant recovery in sensorimotor function after MSC treatment. In all three treatment groups (1 × 10^6^ autoMSCs; 2.5 × 10^6^ autoMSCs; 5 × 10^6^ autoMSCs, *n* = 6 each) there was a significant decrease in mNSS at 7, 30 and 60 days after treatment in individual rats as compared to their own pre-implantation scores (day 0 = 100% total deficit) unlike the small degree of spontaneous recovery seen in MCAO only and MCAO plus PBS controls. (**B**) The same significant recovery of function over time was evidenced when absolute mNSS scores for each MSC treatment group were compared directly to MCAO only and MCAO+PBS control groups. Data are presented as the Standard Deviation (SD) from the mean. *P ≤ 0.05, **P≤0.01, ***P≤0.001, ****P≤0.0001

As expected, after a large cortical and subcortical stroke, there was a significant acute decline in sensorimotor function, with the mean score in infarcted animals being 10.9/16 at 1 day following MCAO with stabilization over the next month (Supplementary Fig. 2A). Importantly however, following transplantation of autoMSCs into the chronic stroke brain, there was a significant recovery of function in all dose groups when compared to themselves or to the small degree of spontaneous behavioral recovery observed over the next 2 months in MCAO-only or MCAO+PBS controls. Thus, when compared to their pre-implantation scores, in the MCAO + 1 × 10^6^, 2.5 × 10^6^, 5 × 10^6^ autoMSC groups, a recovery of 33%, 36%, and 31% was observed at 7 days which rose to 70%, 67%, and 54%, respectively by day 60 post-implantation unlike the small degree of spontaneous recovery seen in MCAO only (29.2%) and MCAO plus PBS controls (30%). When absolute mNSS scores for each MSC treatment group were compared directly to MCAO only and MCAO+PBS control groups, the same significant recovery of function over time was evidenced. Interestingly, there was no dose-dependency observed with MSC treatment, possibly indicating that autoMSCs in all dose groups exceeded a threshold for treatment efficacy (Fig. 1).

### Change in Infarct Volume using Magnetic Resonance Imaging

**Fig. 2:**
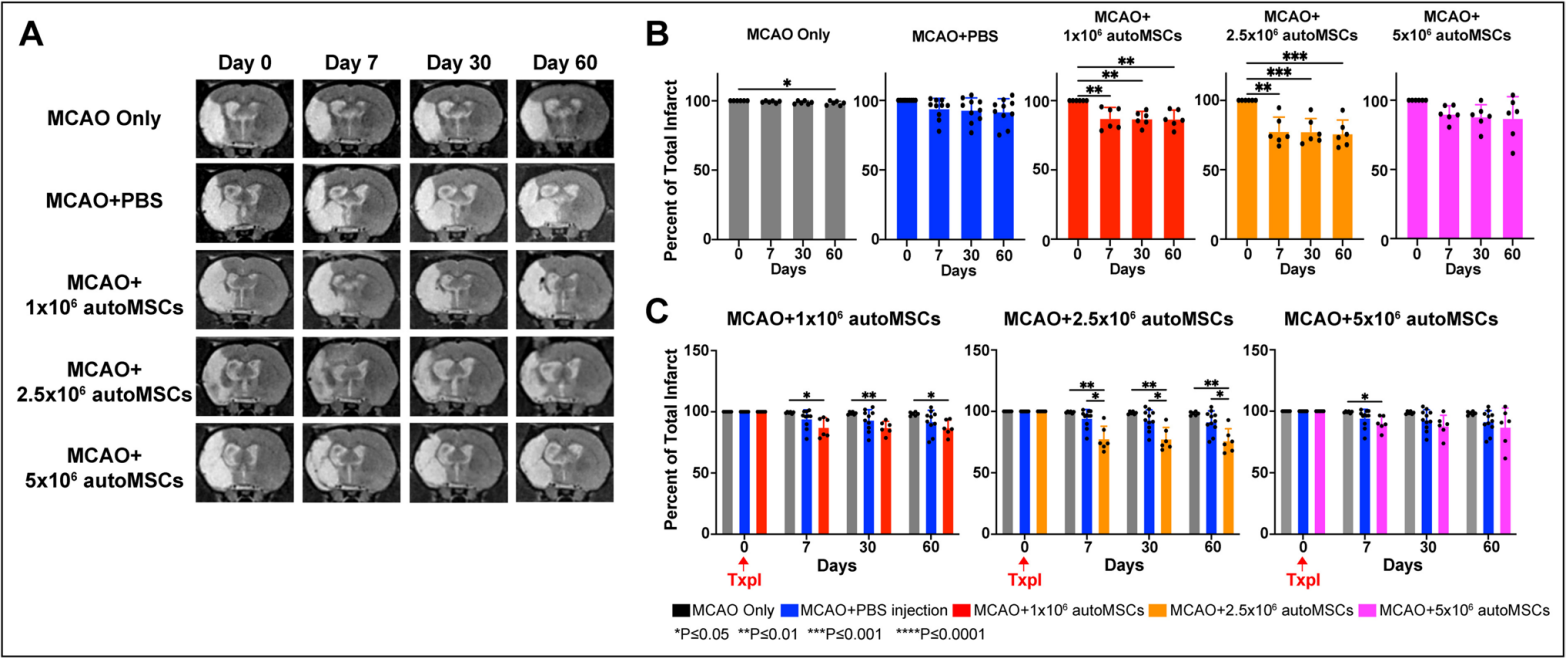
Minor changes in MCAO volume after autoMSC transplantation into the chronic stroke brain. (**A**) T2-Weighted MR images showing MCAO only and MCAO+PBS controls MCAO+1 × 10^6^, MCAO+2.5 × 10^6^, MCAO+5 × 10^6^ autoMSC treatment groups at 0-, 7-, 30-, and 60 days post-transplantation. (**B**) As each infarct is different, infarct volume was measured in individual rats, and changes in volume due to treatment were compared to pre-implantation measurements (day 0 =100%). Percent change in infarct size reveals a small but significant change to infarct size following injection of stem cells in MCAO+1 × 10^6^ autoMSCs and MCAO+2.5 × 10^6^ autoMSCs groups (*n* = 6 each) at 7, 30 and 60 days when compared to pre-implantation size (day 0 =100%). MCAO+5 × 10^6^ autoMSCs group (*n* = 6) trended down but showed no significant change in infarct size. (**C**) MCAO only (*n* = 6) and MCAO+PBS controls (*n* = 9) infarct size did not differ significantly from each other but when compared to MCAO+1 × 10^6^ autoMSCs and MCAO+2.5 × 10^6^ autoMSCs groups, small but significant decreases in infarct volume were observed. While the MCAO+5 × 10^6^ autoMSCs group trended down, the decline did not reach statistical significance. Data are presented as the Standard Deviation (SD) from the mean. *P≤0.05 **P≤0.01 ***P≤0.001 ****P≤0.0001

To assess infarct volume changes as a result of MSC administration, MRI was utilized. Infarct volume was measured one-day following MCAO (day 28), on the day of autoMSC injection (day 0), as well as 7-, 30-, and 60 days following injection. We found that the edema and swelling observed 1 day after stroke subsided by the day of cell implantation 28 days later (Supplementary Fig. 2B**)**. Stroke lesion size remained relatively constant over the next 60 days in the control groups **(**Fig. 2A**)**. The MCAO-only control group had the smallest change in infarct size, decreasing by 1% while the MCAO+PBS control group decreased by 9% over 60 days (Fig. 2B**)**. In contrast, when compared to their pre-implantation stroke volume, the rats treated with 1 × 10^6^ autoMSCs saw a small but significant 13% decrease in infarct size by 7 days post-transplantation while the MCAO+2.5 × 10^6^ autoMSCs group decreased by 23%, both of which remained constant over the next 60 days. Changes in infarct volume in the MCAO+5 × 10^6^ autoMSCs trended downward but did not reach significance **(**Fig. 2C**)**. Absolute values for infarct volume are provided in Supplementary Figure 3. In this case, where the groups of cell-treated rats were compared to control groups, the small effects of autoMSCs were lost due to the variability in infarct volume from one stroke to another, thus highlighting the value of studying individual rats where changes in stroke volume due to MSCs can be compared pre- and postimplantation.

Additionally, it has been previously reported that repeated doses of intravenous (IV) MSCs results in increased thickness of the corpus callosum (CC) [52]. To analyze this parameter in our study, 25 μm axial cryosections were stained with cresyl violet and measured the width of the corpus callosum along the midline at bregma -0.26 (Supplementary Figure 4A). Average CC width for the MCAO-only group was 0.92 mm and MCAO+PBS group was 0.77 mm, while that in MCAO + 1 × 10^6^, 2.5 × 10^6^, 5 × 10^6^ autoMSC groups was 0.86 mm, 0.82 mm, and 0.86 mm respectively. Thus, no significant differences existed in average CC width between experimental groups (Supplementary Figure 4B).

### In-Vivo Tracking of Q-Dot labeled autoMSCs

To assess location and migratory behavior of transplanted autoMSCs, cells were labeled with quantum dot nanoparticles (Q-dots). In a separate study, bone marrow was aspirated from the rat and adherent autoMSCs were grown in flasks as described above. Q-dots were added to media 24 h before transplantation and allowed ample time to endocytose into the cells. Cells were then harvested after 24 h, followed by repeated rinses to remove unbound Q-dots, resuspended to their final concentration (2.5 × 10^6^ autoMSCs) and transplanted into the rat peri-infarct area as described above. Animals were sacrificed at day 7 (*n* = 3), day 30 (*n* = 3), and day 60 (*n* = 3) to determine cell localization over time.

**Fig. 3:**
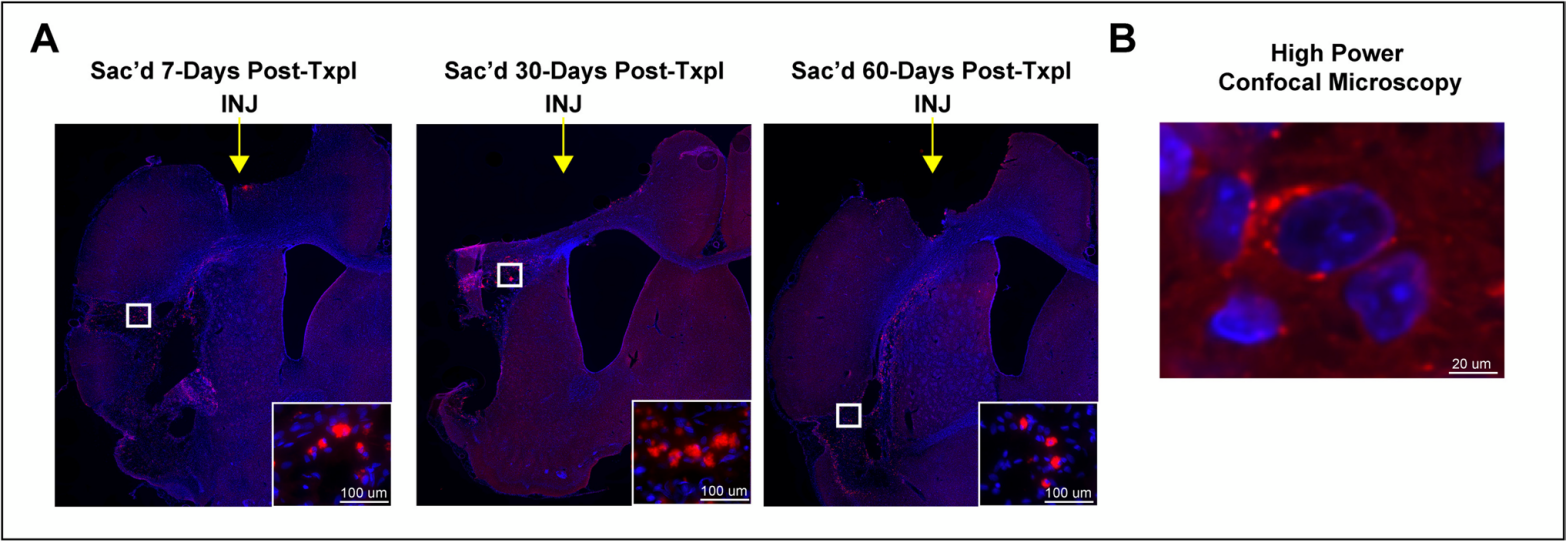
AutoMSCs remain at the transplantation site long-term. (**A**) Photomicrographs revealing the presence of quantum dots restricted to the area of the injection in the peri-infarct region at day 7-, 30-, and 60 days post-transplantation, with no spread of labeled autoMSCs to other brain regions. (**B**) High power confocal microscopy shows the presence of quantum dots (red) within the cytoplasm around the DAPI-stained nucleus (blue)

Following sacrifice, brains were removed and cryosectioned (25 μm coronal sections) and subsequently counterstained with DAPI. Since the stereotactic apparatus used to place cells in the brain is not directly integrated with the MRI, we cannot precisely position cells in the peri-infarct region. Consequently, variable numbers of implanted cells are lost in the infarct core, making quantification of cell survival and comparisons from animal to animal impractical. However, qualitatively, Q-dot labeled MSCs remained in the brain long term (60 days post-transplantation) and stayed localized to the peri-infarct site where they had been implanted without migration elsewhere in the brain over time (Fig. 3).

### Long-Term Grafts Exhibit No Tumorigenesis

**Fig. 4:**
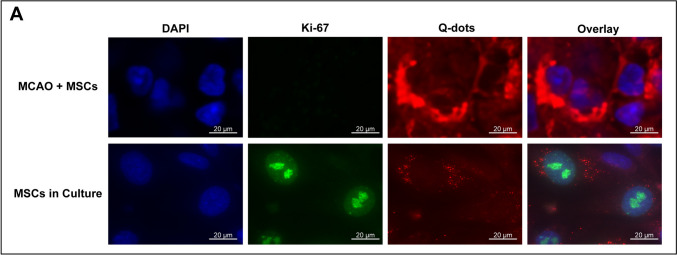
Absence of dividing Ki-67 aMSCs at 60 days post-transplantation. Fluorescence confocal microscopy of an MCAO rat brain showing the lack of Ki-67 (green) nuclear staining in transplanted aMSCs whose cytoplasm was labeled with Q-dots (red), indicating the absence of cell division at 60 days post transplantation. In contrast, dividing MSCs grown in culture show abundant Ki-67 nuclear staining in Q-dot labeled cells, indicating that Q-dots do not themselves interfere with cell division

**Fig. 5:**
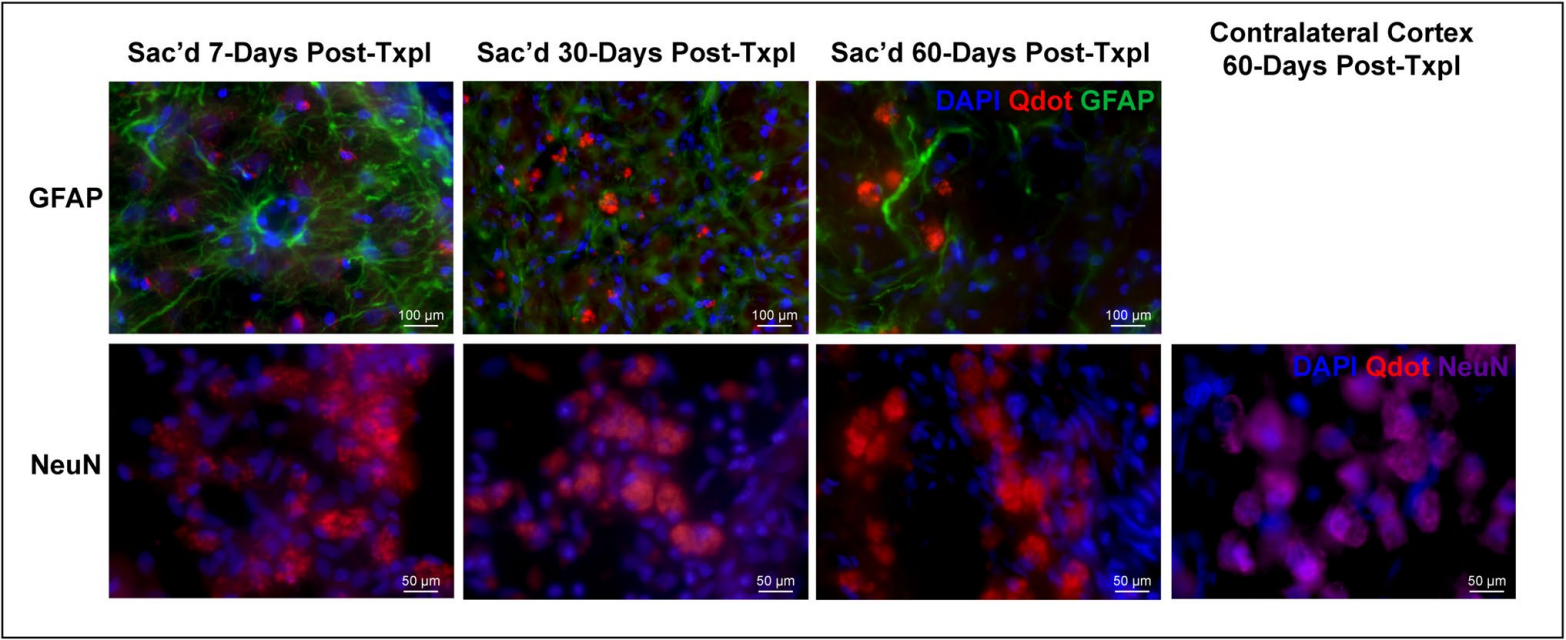
AutoMSCs do not differentiate or transdifferentiate into neurons or astrocytes following transplantation into the chronic stroke brain. Immunofluorescent staining of GFAP (reactive astrocytes) and NeuN (neurons) at day 7-, 30-, and 60- post-transplantation reveals no overlap in Q-dot- labeled aMSCs, although positive staining for NeuN on the contralateral side is evident

**Fig. 6:**
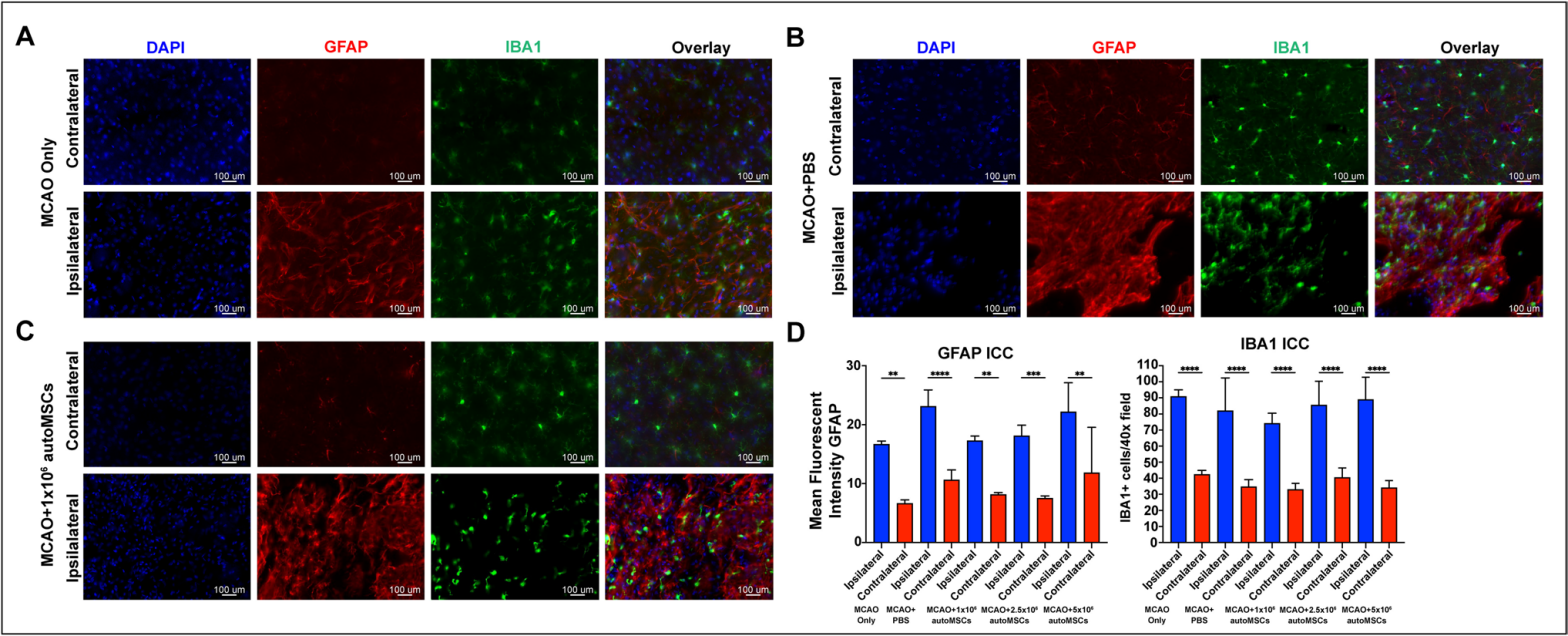
Long-term astrocyte and microglial reactivity on the side ipsilateral to MCAO with no change from autoMSC treatment. (**A**–**C**) Immunofluorescent staining of GFAP (reactive astrocytes) and IBA1 (reactive microglia) at 60 days post-transplantation reveals sustained reactive gliosis in the chronic stroke brain treated PBS or autoMSCs, and without treatment. (**D**) Quantification of GFAP mean fluorescent intensity in astrocytes per 40× field and number of IBA1 positive microglia per 40× field, indicating that stroke increases glial reactivity on the ipsilateral (blue bar) compared to the contralateral side (red bar) but does not change further with 1 × 10^6^, 2.5 × 10^6^ or 5 × 10^6^ autoMSC treatment. Likewise, when compared to MCAO+PBS controls (red bar), autoMSC treatment at all cell doses (blue bars), there were no significant differences in gliosis on either the ipsilateral or contralateral sides. Data are presented as mean plus/minus standard error mean (SEM). *P≤0.05 **P≤0.01 ***P≤0.001 ****P≤0.0001

Given the persistence of autoMSCs in the brain, investigation of the cells’ tumorigenic potential over time was necessary. There was no evidence of tumor-like structures in MRI images (Fig. 2A), or upon histological examination **(**Fig. 3, 5, 6**)**. Q-dot transplanted brains were also stained with antibodies to the cell cycling protein Ki-67, a widely used marker of tumor cell growth. Cryosections were prepared as described in Methods and stained for Ki-67 and DAPI. No Ki-67 staining in Q-dot labeled cells was observed in any of the 9 rats examined at 3 time points. In contrast, Ki-67 robustly stained rapidly proliferating MSCs in culture as a control (Fig. 4).

### MSCs Do Not Transdifferentiate In-Vivo

Since intracerebrally injected autoMSCs remain in the brain long term, we sought to determine the downstream fate of autoMSCs following transplantation in-vivo. Q-dot brains were cryosectioned as previously described and stained for the neuronal markers NeuN, as well as the reactive astrocyte marker Glial Fibrillary Acidic Protein (GFAP). We found no transdifferentiation of our Q-dot labeled autoMSCs into neurons or astrocytes at 7-, 30-, and 60 days (Fig. 5).

### Mesenchymal Stem Cell Effects on Reactive Gliosis

It has previously been shown that MSCs produce significant changes in the immune landscape of the brain following transplantation into the ischemic brain [53]. Therefore, following sacrifice at 60 days post-MCAO, cryosections were stained for the reactive astrocyte marker, Glial Fibrillary Acidic Protein (GFAP), and the reactive microglia marker Ionized Calcium Binding Adaptor Molecule 1 (IBA1) (Fig. 6A). As expected, after MCAO, there was a highly significant increase in mean fluorescent intensity of GFAP, and a highly significant increase in the number of IBA1+ cells in the peri-infarct region, when compared to the contralateral side of the brain (Fig. 6B). Cells remained in the activated state even 60 days after MCAO, and importantly, these parameters were not modified by the presence of autoMSCs in the brain.

### Allogeneic Mesenchymal Stem Cells

**Fig. 7:**
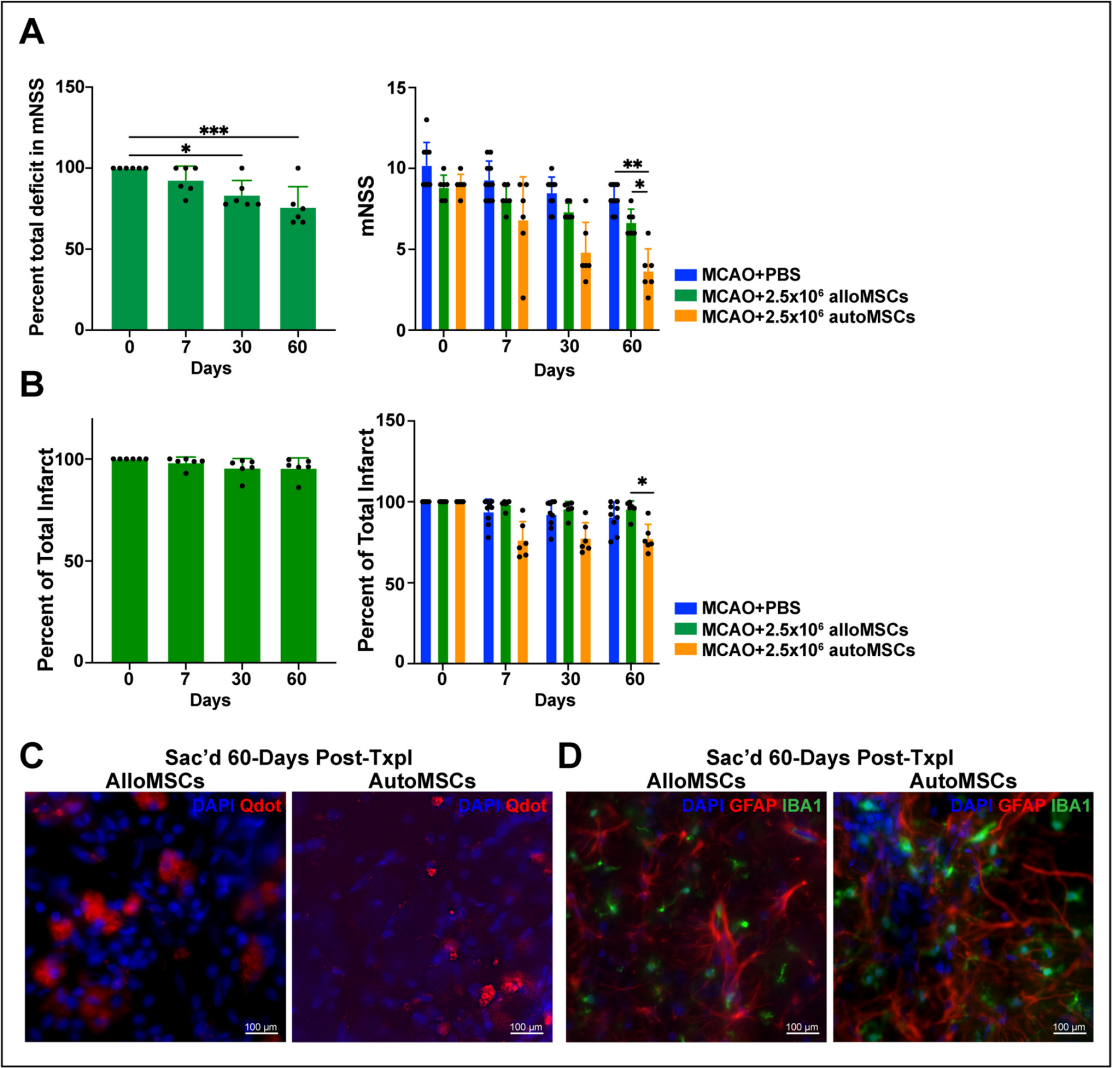
The effects of AlloMSC transplantation into a chronic stroke rat. (**A**) Behavioral assessments after alloMSC transplantation in individual alloMSC-grafted rats (green bars) revealed small but significant decreases in mNSS over time when compared to their own pre-implantation scores (day 0 = 100% total deficit). However, this improvement did not differ significantly from the spontaneous recovery seen in MCAO+PBS control animals (blue bars) and was far less than that seen after autoMSC transplantation (gold bars). (**B**) In contrast to autografts, allografts did not decrease infarct size. (**C**) Photomicrographs of labeled alloMSCs or autoMSCs revealing the presence of quantum dots restricted to the area of the injection in the peri-infarct region at 60 days post-transplantation, with no spread of labeled alloMSCs to other brain regions. (**D**) Immunofluorescent staining of GFAP (reactive astrocytes) and IBA1 (reactive microglia) at 60 days post-transplantation revealed sustained reactive gliosis in the chronic stroke brain treated with alloMSCs or autoMSCs. Data are presented as the Standard Deviation (SD) from the mean. *P≤0.05 **P≤0.01 ***P≤0.001 ****P≤0.0001

Since nearly all published studies on MSC therapies in stroke utilize allogeneic MSCs derived from the bone marrow of healthy rats, we examined the effects of these cells (2.5 × 10^6^ alloMSCs) transplanted into immunosuppressed (cyclosporin-treated) rats with a chronic 28-day old MCAO stroke and compared these results to our findings with autoMSCs. We found that unlike autoMSCs, alloMSCs produced only small improvements in sensorimotor function (31.7%) that were not significantly different from the spontaneous recovery seen in MCAO + PBS control animals (30%) with no significant reduction in stroke volume (Fig. 7A, B; absolute values are provided in the Supplementary Figure 5) or change in corpus width (Supplementary Figure 4). This occurred despite the fact that alloMSCs remained in the graft at 60 days post-transplantation surrounded by highly reactive glia from the stroke (Fig. 7C, D).

## Discussion

Currently, there are no approved treatments for chronic ischemic stroke patients, having been woefully understudied. Until now, stem cells have been thought to hold potential for improving recovery after stroke by modifying neuroinflammation during the acute phase of ischemia when most cells are lost in the infarct core, or during the subacute phase when more plasticity is observed in the peri-infarct region. Quite remarkably, in this study, however, we found that autologous MSCs transplanted directly into the brain of rats with a chronic infarct steadily enhanced recovery of function over the subsequent months, demonstrating effects long after typical windows of plasticity in rats or stroke patients.

Many studies in rodents and non-human primates have previously shown the benefits of MSC treatment in improving functional recovery in acute ischemic stroke models [14–31, 35, 38, 40, 41, 54–56]. With the exception of a few studies that used autoMSC [35, 40], most investigations have employed alloMSCs delivered systemically [41, 54–56] or via direct intracerebral injection [56–64]. Thus far, very few studies have examined MSC treatment during the chronic phase of stroke, and all of these have used IV delivered alloMSCs [32–34].

Our current study investigated the use of autologous MSCs injected intracerebrally in a chronic stroke rat model. A major advantage of our approach over many previously published animal studies, is the attempt to adhere to the STEPS guidelines for stem cell therapies for stroke (i.e., dose response analysis, timing of cell delivery, etc.) [14, 51]. In addition, wherever possible, we have harmonized preclinical procedures with those that might be practicable in the clinic such as the use of a chronic stroke model that allows for the expansion of autologous MSCs and their subsequent intracerebral transplantation, which would not be feasible in an acute setting. Also, this study used intracerebral injections rather than systemic MSC injections that can result in microembolism [65]; also MRI-guided cell delivery and individualized cell placement to better approximate clinical practices.

We showed that at all tested cell doses (1 × 10^6^, 2.5 × 10^6^, 5 × 10^6^ autoMSCs), rats gained significant functional recovery measured by behavioral testing scores. Moreover, we found that recovery in sensorimotor function began within 1 week of autoMSC administration and continued to improve significantly over the next 60 days. The absence of dose dependency seen in these studies has been well documented previously [62] and suggests that there is a minimally effective dose which is exceeded even in our lowest tested cell concentration (1 × 10^6^ autoMSCs). It will be important in the future to determine the lower limit in cell dosage needed to achieve therapeutic efficacy. The striking recovery in behavior following intracerebral autoMSC transplantation indicates the positive and long-lasting effects of direct injection of stem cells into the peri-infarct region, even in a chronic stroke after the window of brain plasticity is presumed to have closed.

Interestingly, in our study and other studies using a chronic stroke model, no significant improvement in mNSS, rotorod or wire hang testing [33, 34] was noted until MSCs were implanted. This is in contradistinction to the spontaneous recovery of sensorimotor behaviors reported in the acute stroke model [14–31, 35, 38, 40, 41, 54–56]. This discrepancy may be related to a number of differences in the chronic stroke model, such as long term secondary effects on the brain like the degeneration of corticothalamic connections [66]. Another possible explanation for this difference is that behavior was not regularly evaluated during the first month while the chronic infarct was developing in our study, whereas in the acute model, rats were tested at multiple time points during the first month, essentially subjecting animals to a form of physical rehabilitation during the subacute phase, a period of heightened plasticity. This “unintended physical therapy” may benefit control animals, improving spontaneous behavioral recovery and may further enhance functional recovery after cell transplantation. The effects of early rehabilitation on MSC transplantation in a chronic stroke model has not yet been investigated.

There remains inconclusive evidence regarding the effect that MSC administration has on infarct size [35, 57, 62, 67]. In investigating changes to the volume of the stroke after autoMSC administration, it must be noted that MR imaging revealed a much larger infarct on the day following MCAO due to cerebral edema. This acute swelling of the brain subsided by the day of transplantation at which time stroke volume had stabilized in control rats. Nonetheless, in the two lower dose autoMSC treatment groups we found a small but significant reduction in infarct volume when compared with control groups. The higher dose group (MCAO+5 × 10^6^ autoMSCs) trended down but did not reach significance, possibly due to the small sample size of the experimental groups or variability in the placement and survival of implanted MSCs. The small graft-associated changes in infarct volume were unsurprising given that cell death in the ischemic core had likely equilibrated by the end of the chronic phase (first 28 days) prior to MSC transplantation. Likewise, we found that the width of the corpus callosum was also unchanged following transplantation in all treatment groups when compared to controls. This differs from previous research in acute stroke models showing that single and repetitive MSC treatment increases the thickness of the corpus callosum, suggestive of regrowth of myelinated fibers and synaptic plasticity [52]. Possibly, an older more chronic stroke does not lend itself to this type of brain recovery.

We further used Q-dot-labeled autoMSCs to track the localization of MSCs in the brain over time. We found that labeled implanted cells remained at the locations in the peri-infarct region where they had been originally deposited, without migration elsewhere in the brain, even two months later. The lack of cell migration may result from the glial scar that forms around deposited cells as described by others [68]. It is likely that this long-term survival of autoMSCs and the continual availability of their locally secreted products may have critically contributed, either directly or indirectly, to the observed robust long-term recovery in sensorimotor function seen in this chronic stroke model.

Although the use of stem cells raises a concern of potential tumorigenicity due to their innate ability to self-renew, we found no evidence of cell proliferation after implantation of autoMSCs as evidenced by the lack of Ki-67 in and around the transplanted region at two months. Furthermore, we did not observe abnormal tumor-like anatomy on MRI at any timepoint during the study. Interestingly, while there was no indication that MSCs were dividing in the graft, there was also no evidence that cells had differentiated into other brain phenotypes. Thus, NeuN or GFAP staining was not seen in Q-dot-labeled transplanted MSCs, indicating the absence of differentiation or trans-differentiation of MSCs into neurons and glia.

Of possible further importance is the local cellular and inflammatory landscape into which MSCs were transplanted. Indeed, we demonstrated a sustained increase in the degree of reactivity in astrocytes and in the number of reactive microglia in all rats with a large MCA stroke which was unaltered by MSC therapy, though potential changes in their molecular composition (i.e., cytokines, growth factors, etc.) were not studied here. The literature on reactive gliosis following stem cell transplantation in rats with ischemia is conflicted with some studies showing an increase and others a decrease [37, 38, 41, 53, 60]. Regardless, the persistent glial reactivity seen after stroke may reflect critical changes in the local cellular and molecular milieu needed for autoMSCs to produce enhanced functional recovery in this chronic rat stroke model.

Finally, in a separate important study, we directly compared our results using autoMSCs with alloMSCs in the chronic stroke model. Interestingly, in immunosuppressed rats, alloMSCs survived long term in the brain similar to autoMSCs. However, unlike autografts, allografts did not produce functional recovery greater than the spontaneous recovery recorded in control animals. This is in agreement with the observations of others using alloMSCs [33, 34] despite a report of improved blood-brain-barrier (BBB) function in these rats [32].

In our study, the disparity between the efficacy of autoMSCs versus alloMSCs may be due to small differences in cell handling (i.e., autoMSCs but not alloMSCs were expanded in the bioreactor) or cell survival in the graft (i.e., alloMSCs may be subject to greater immunorejection than autoMSCs). However, more likely, the key difference stems from the fact that autoMSCs were harvested from the bone marrow of a rat with an active stroke while alloMSCs, as in all allografts studied previously in stroke [16–27, 29–34, 37, 38, 41, 54–58, 62, 67, 69–73], were derived from a healthy (non-MCAO) donor rat. This critical difference, which likely impacts the profile of cytokines and growth factors autoMSCs secrete into their environment, may be crucial to treatment efficacy. Possibly alloMSCs, which are known to be most effective when administered soon after stroke [40, 74], are provided this critical activation in the acute stroke model but not in the chronic stroke model unless combined with other potentially activating influences, like rehabilitation therapy [33, 34]. Consistent with this notion, alloMSCs that had been genetically engineered and transplanted as a “modified stem cell product”, and thus potentially activated, proved partially effective in a preliminary clinical trial of chronic stroke patients [15, 28]. Resolving these important underlying mechanisms will require further exploration into the molecular crosstalk between local brain cells and implanted MSCs from various sources. Regardless of the mechanisms, the results of the current study in rats have important clinical implications, suggesting that additional recovery in patients with chronic infarcts and long-term disability may be possible with intracerebral autoMSC therapy.

## Supplementary information

ESM 1 Supplemental Figure 1: Study design overview. Supplemental Figure 2: Analysis of the chronic stroke model. (A) Pooled analysis of mNSS for all rats used in these studies, highlighting that after a large ischemic cortical and subcortical stroke, there was a significant acute decline in sensorimotor function, with the mean score in infarcted animals being 10.9/16 at one day following MCAO with stabilization to 9.5/16 over the next month prior to treatment. (B) Pooled analysis of infarct volume for all rats used in these studies, highlighting that after a large ischemic cortical and subcortical stroke, there was a significant decline in infarct volume due to acute edema. (C) Representative T2-Weighted MR images showing the change in edema associated with the transition from an acute stroke to a chronic stroke. *P≤0.05 **P≤0.01 ***P≤0.001 ****P≤0.0001. Supplemental Figure 3: Changes in MCAO volume after autoMSC transplantation into the chronic stroke brain. When absolute values for infarct volume between controls MCAO only (n=6) and MCAO+PBS controls (n=9) are compared to MCAO+1x10^6^, MCAO+2.5x10^6^ or MCAO+5x10^6^ autoMSCs groups, the relatively small decreases in infarct volume seen in Fig. 2B/C (infarcts are normalized to themselves pre- and post-implantation) disappears due to the variability in individual strokes. Data are presented as the Standard Deviation (SD) from the mean. Supplemental Figure 4: No significant changes to corpus callosum width with aMSC treatment. (A) Representative images showing cresyl violet stained brains at bregma -0.26mm from MCAO only, MCAO+PBS, MCAO+1x10^6^, MCAO+2.5x10^6^, MCAO+5x10^6^ autoMSCs, and MCAO+2.5x10^6^ alloMSC groups, highlighting the corpus callosum (black box). (B) Quantification of corpus callosum width reveals a small but significant difference when comparing MCAO only and MCAO+PBS control groups, with no significance between experimental groups. Data are presented as mean plus/minus standard error mean (SEM). *P≤0.05 **P≤0.01 ***P≤0.001 ****P≤0.0001. Supplemental Figure 5: The effects of AlloMSC transplantation into a chronic stroke rat. When absolute values for infarct volume between MCAO+PBS controls (n=9) are compared to MCAO+2.5x10^6^ autoMSCs or MCAO+5x10^6^ alloMSCs groups (n=6 each), the relatively small decrease in infarct volume seen in Fig. 7B (infarcts are normalized to themselves pre- and post-implantation) disappears due to the  variability in individual strokes. Data are presented as the Standard Deviation (SD) from the mean. Supplemental Table 1: Summary of Treatment-Emergent Adverse Events in Rats Following Intracerebral Transplantation of aMSCs. Supplemental Table 2: Decreased Appetite and Body Weight Lost Following Surgery. Supplemental Table 3: Modified Neurological Severity Score (DOCX 2135 kb)
