# Supplementary material for: Intracerebral Transplantation of Autologous Mesenchymal Stem Cells Improves Functional Recovery in a Rat Model of Chronic Ischemic Stroke
Source: Transl Stroke Res. 2023 Nov 2;16(2):248–61. doi: 10.1007/s12975-023-01208-7 (PMC11976345; doi:10.1007/s12975-023-01208-7)
Supplement: Supplementary file 1 — Supplemental Figure 1: Study design overview. Supplemental Figure 2: Analysis of the chronic stroke model. (A) Pooled analysis of mNSS for all rats used in these studies, highlighting that after a large ischemic cortical and subcortical stroke, there was a significant acute decline in sensorimotor function, with the mean score in infarcted animals being 10.9/16 at one day following MCAO with stabilization to 9.5/16 over the next month prior to treatment. (B) Pooled analysis of infarct volume for all rats used in these studies, highlighting that after a large ischemic cortical and subcortical stroke, there was a significant decline in infarct volume due to acute edema. (C) Representative T2-Weighted MR images showing the change in edema associated with the transition from an acute stroke to a chronic stroke. *P≤0.05 **P≤0.01 ***P≤0.001 ****P≤0.0001. Supplemental Figure 3: Changes in MCAO volume after autoMSC transplantation into the chronic stroke brain. When absolute values for infarct volume between controls MCAO only (n=6) and MCAO+PBS controls (n=9) are compared to MCAO+1x10^6^, MCAO+2.5x10^6^ or MCAO+5x10^6^ autoMSCs groups, the relatively small decreases in infarct volume seen in Fig. 2B/C (infarcts are normalized to themselves pre- and post-implantation) disappears due to the variability in individual strokes. Data are presented as the Standard Deviation (SD) from the mean. Supplemental Figure 4: No significant changes to corpus callosum width with aMSC treatment. (A) Representative images showing cresyl violet stained brains at bregma -0.26mm from MCAO only, MCAO+PBS, MCAO+1x10^6^, MCAO+2.5x10^6^, MCAO+5x10^6^ autoMSCs, and MCAO+2.5x10^6^ alloMSC groups, highlighting the corpus callosum (black box). (B) Quantification of corpus callosum width reveals a small but significant difference when comparing MCAO only and MCAO+PBS control groups, with no significance between experimental groups. Data are presented as mean plus/minus standard error mean (SEM). *P≤0.05 **P≤0.01 ***P≤ [file 12975_2023_1208_MOESM1_ESM.docx]

Supplementary Materials

**
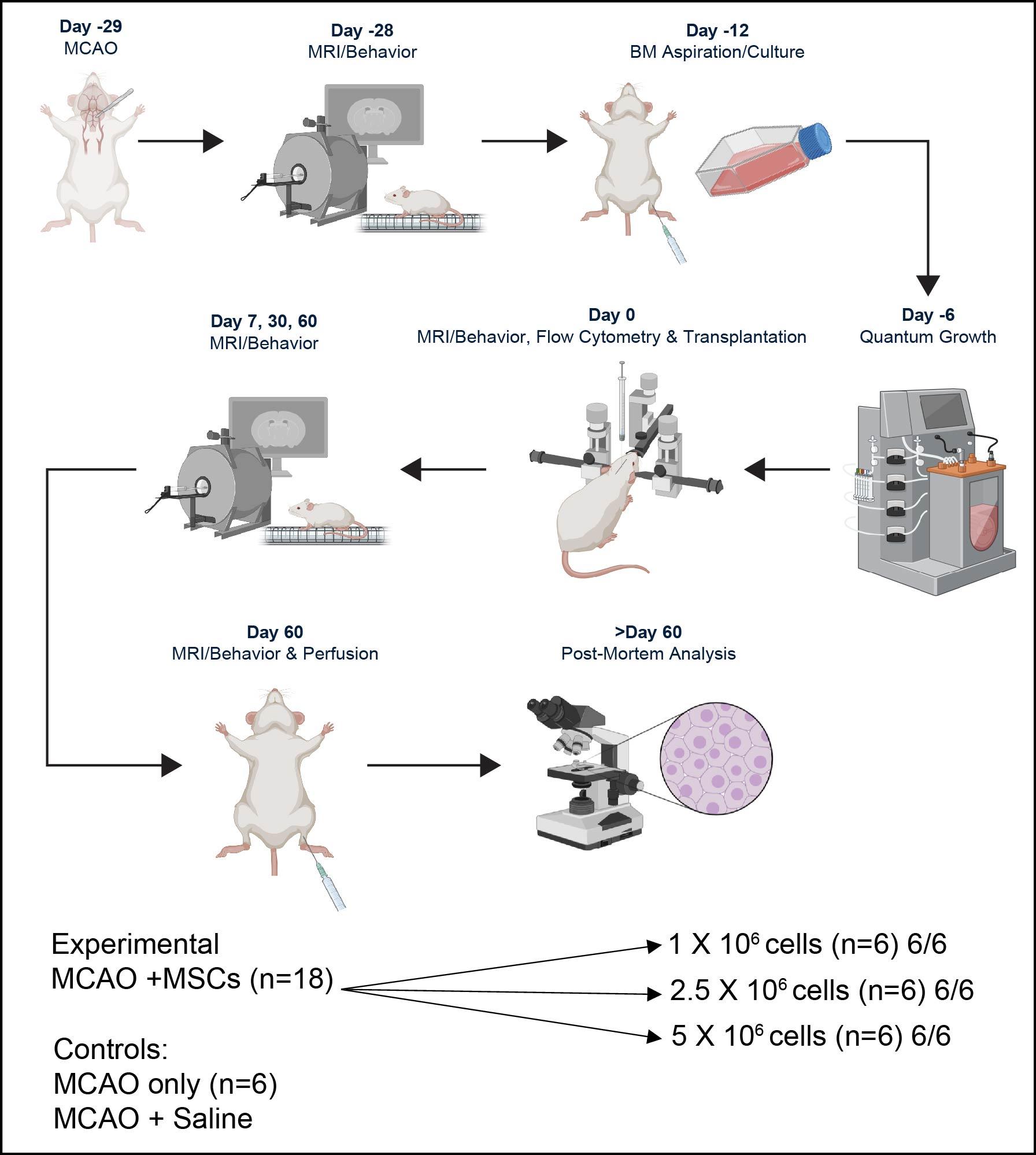
**

**Supplemental Figure 1:** Study design overview.

**
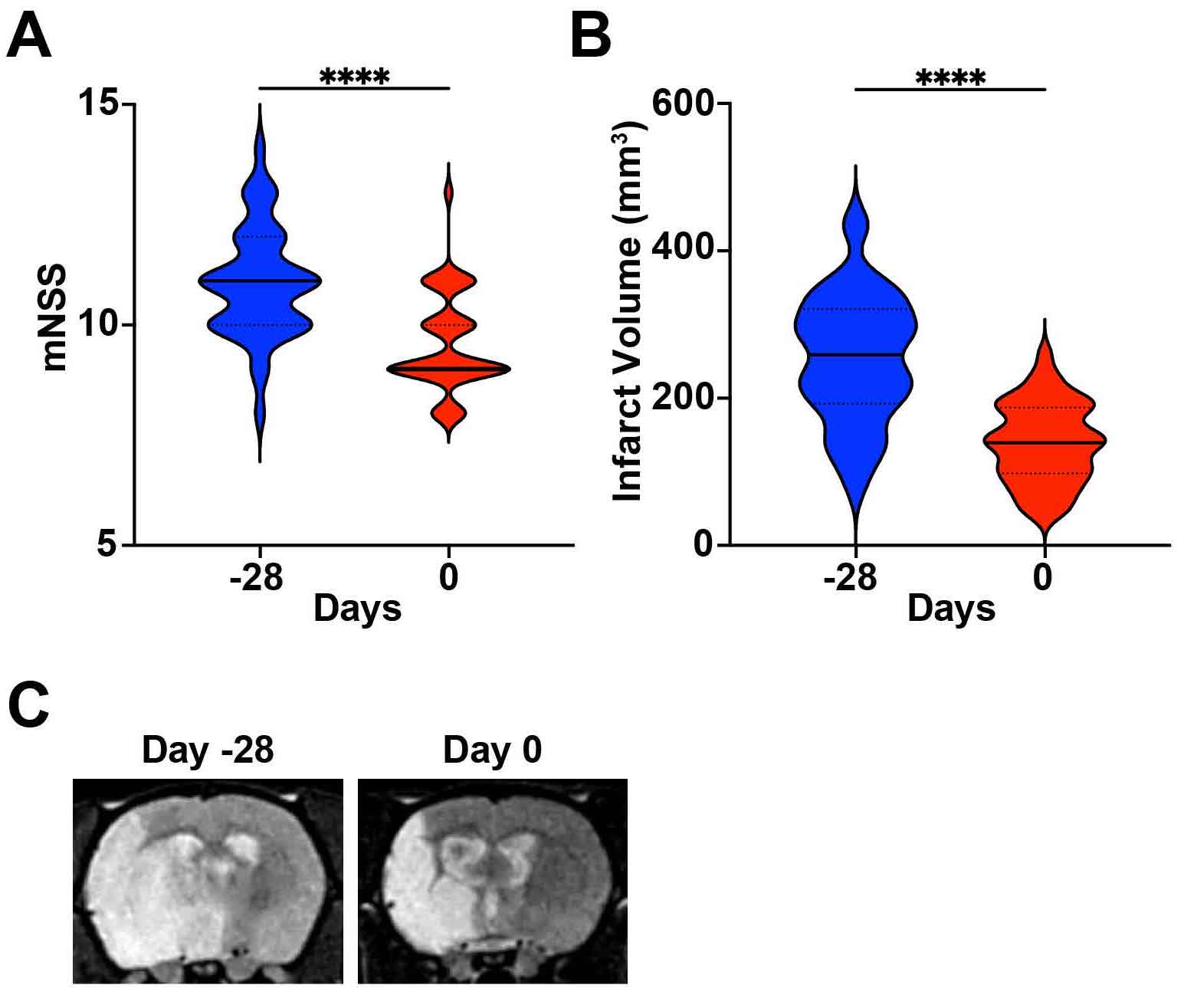
**

**Supplemental Figure 2: Analysis of the chronic stroke model.** **(A)** Pooled analysis of mNSS for all rats used in these studies, highlighting that after a large ischemic cortical and subcortical stroke, there was a significant acute decline in sensorimotor function, with the mean score in infarcted animals being 10.9/16 at one day following MCAO with stabilization to 9.5/16 over the next month prior to treatment. **(B)** Pooled analysis of infarct volume for all rats used in these studies, highlighting that after a large ischemic cortical and subcortical stroke, there was a significant decline in infarct volume due to acute edema. **(C)** Representative T2-Weighted MR images showing the change in edema associated with the transition from an acute stroke to a chronic stroke.

*P≤0.05 **P≤0.01 ***P≤0.001 ****P≤0.0001


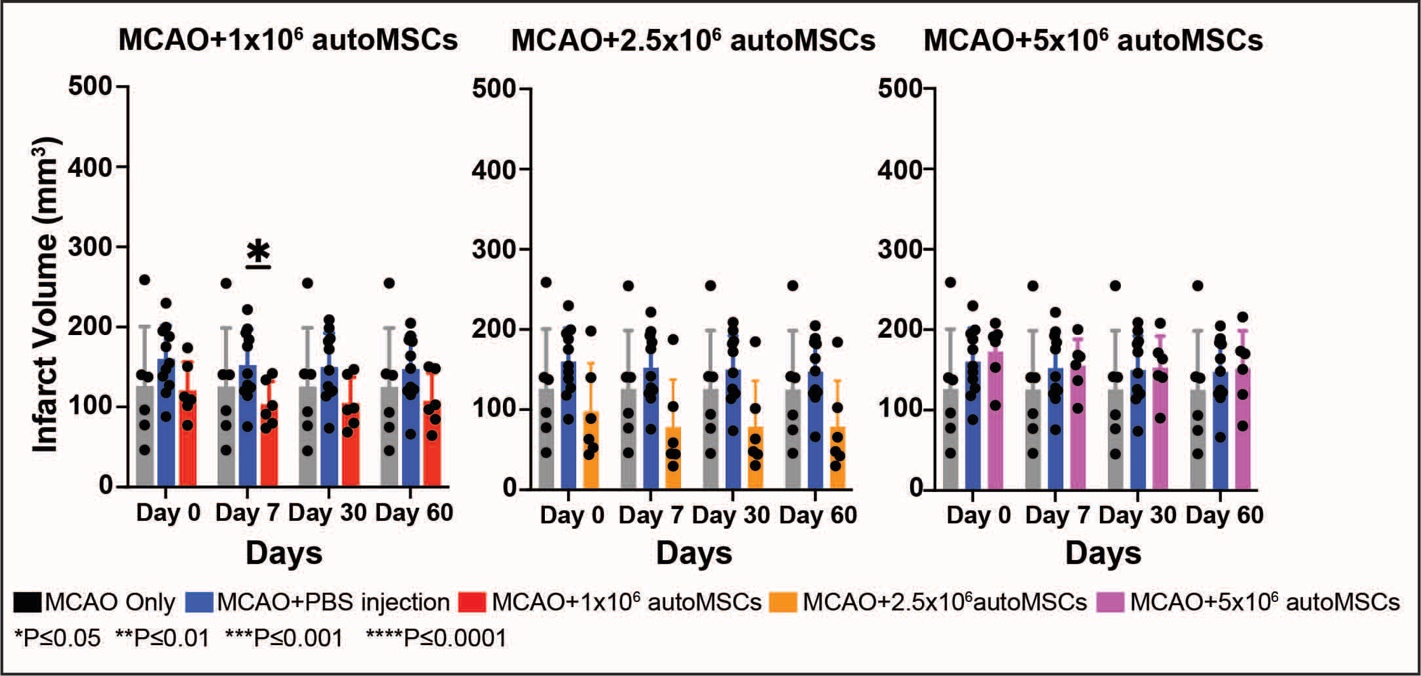
 **Supplemental Figure 3: Changes in MCAO volume after autoMSC transplantation into the chronic stroke brain.** When absolute values for infarct volume between controls MCAO only (n=6) and MCAO+PBS controls (n=9) are compared to MCAO+1x10^6^, MCAO+2.5x10^6^ or MCAO+5x10^6^ autoMSCs groups, the relatively small decreases in infarct volume seen in Fig. 2B/C (infarcts are normalized to themselves pre- and post-implantation) disappears due to the variability in individual strokes.

**
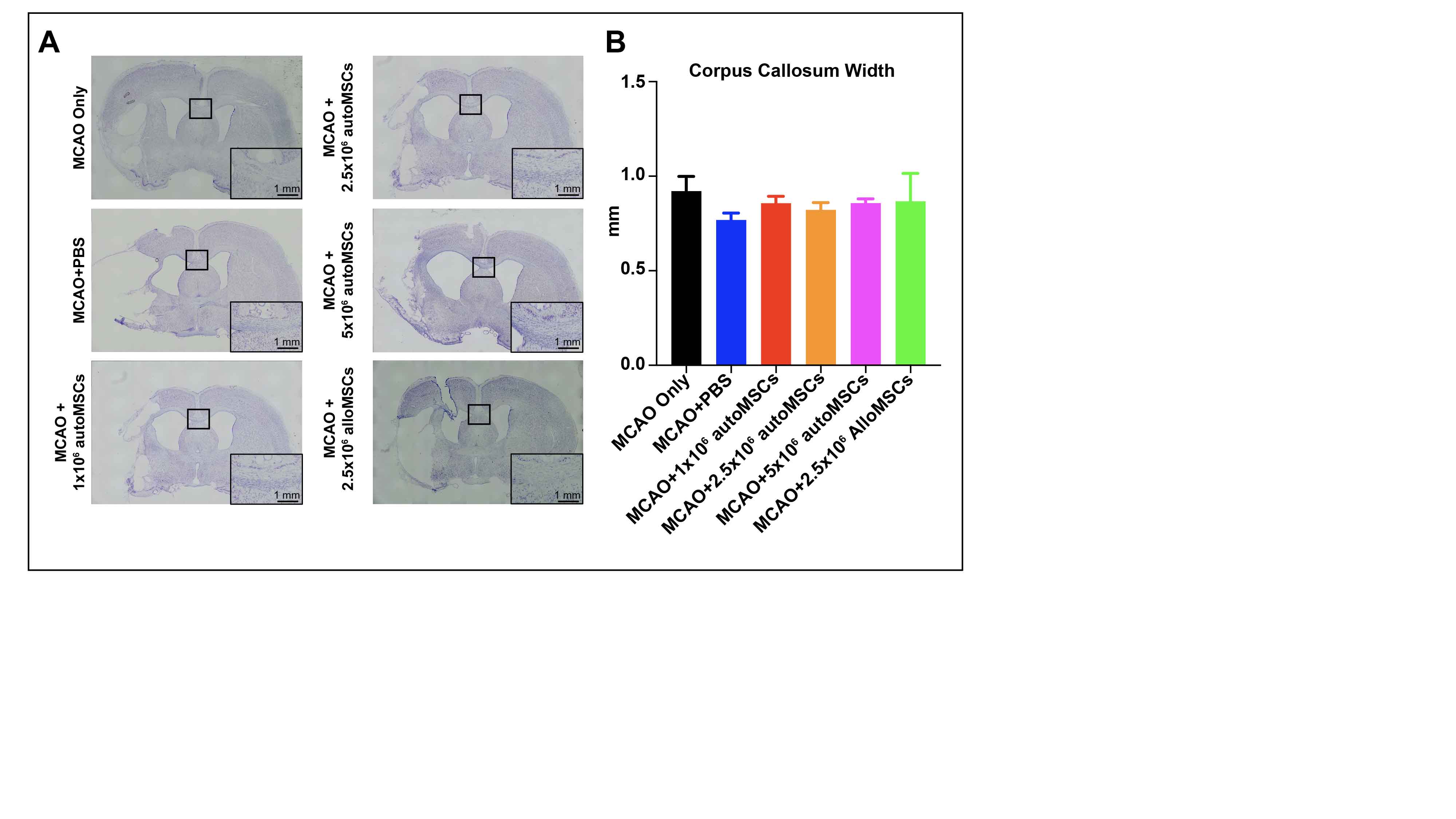
**

**Supplemental Figure 4: No significant changes to corpus callosum width with aMSC treatment. (A)** Representative images showing cresyl violet stained brains at bregma -0.26mm from MCAO only, MCAO+PBS, MCAO+1x10^6^, MCAO+2.5x10^6^, MCAO+5x10^6^ autoMSCs, and MCAO+2.5x10^6^ alloMSC groups, highlighting the corpus callosum (black box). **(B)** Quantification of corpus callosum width reveals a small but significant difference when comparing MCAO only and MCAO+PBS control groups, with no significance between experimental groups. Data are presented as mean plus/minus standard error mean (SEM).

*P≤0.05 **P≤0.01 ***P≤0.001 ****P≤0.0001

**
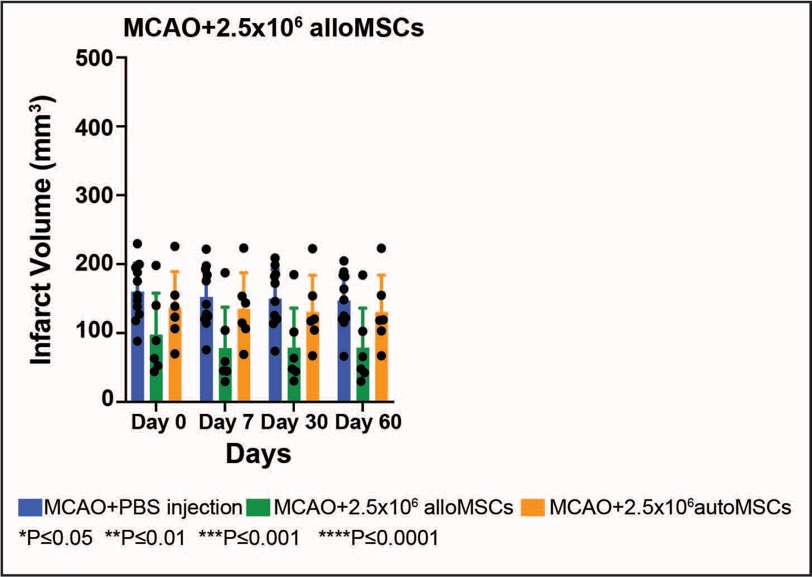
**

**Supplemental Figure 5:** **The effects of AlloMSC transplantation into a chronic stroke rat.** When absolute values for infarct volume between MCAO+PBS controls (n=9) are compared to MCAO+2.5x10^6^ autoMSCs or MCAO+5x10^6^ alloMSCs groups (n=6 each), the relatively small decrease in infarct volume seen in Fig. 7B (infarcts are normalized to themselves pre- and post-implantation) disappears due to the ~~large~~ variability in individual strokes.

| Treatment-Emergent Adverse Events | Result |
| --- | --- |
| Nausea | None |
| Pain | None |
| Decreased appetite | Decreased appetite for 24 hours following aMSCs |
| Diarrhea | None |
| Musculoskeletal pain | None |
| Subdural hematoma | None |
| Seizure | None |
| Infection | None |

**Supplemental Table 1: Summary of Treatment-Emergent Adverse Events in Rats Following Intracerebral Transplantation of aMSCs**

| Animal Identifier | % Bodyweight lost following surgery |
| --- | --- |
| MCAO – 3.1 | None |
| MCAO – 4.3 | 2.8% |
| MCAO – 5.1 | 3.3% |
| MCAO – 6.3 | None |
| MCAO – 8.1 | 1.3% |
| MCAO – 11.3 | 4.4% |
| MCAO – 12.3 | 2.1% |
| MCAO – 13.2 | None |
| MCAO – 14.2 | None |
| MCAO – 15.3 | None |
| MCAO – 17.2 | 3.11% |
| MCAO – 19.2 | None |
| MCAO – 20.11 | None |
| MCAO – 21.5 | 5.7% |
| MCAO – 22.2 | 0.6% |
| MCAO – 23.1 | 9.6% |
| MCAO – 25.2 | None |
| MCAO – 29.2 | 4.8% |
| MCAO – 2.5 | None |
| MCAO – 3.2 | None |
| MCAO – 5.2 | None |
| MCAO – 6.1 | 2.5% |
| MCAO – 8.3 | 5.6% |
| MCAO – 7.5 | 3.2% |
| MCAO – 9.3 | None |
| MCAO – 9.2 | None |
| MCAO – 19.1 | 2.6% |
| MCAO – 30.2 | Animals did not receive transplantation surgery, and therefore did not lose weight. |
| MCAO – 30.3 |  |
| MCAO – 31.1 |  |
| MCAO – 31.3 |  |
| MCAO – 32.1 |  |
| MCAO – 32.2 |  |

**Supplemental Table 2: Decreased Appetite and Body Weight Lost Following Surgery**

| **Description** | **Possible Score** | **Description of Score** |
| --- | --- | --- |
| Postural Signs | 0 | Symmetric forelimb extension when lifted by tail |
|  | 1 | Forelimb flexion only |
|  | 2 | Forelimb flexion and thorax twisting |
| Gait Dysfunction | 0 | Walking straight |
|  | 1 | Walking towards contralateral side |
|  | 2 | Alternate circling and walking straight |
|  | 3 | Alternate circling and walking towards paretic side |
|  | 4 | Circling and/or other gait disturbance |
| Response to Tail Pull | 0 | Symmetric movement |
|  | 1 | Asymmetric movement |
| Proprioceptive Forelimb Placement | 0 | Normal placing |
|  | 1 | Weak or delayed (<2s) placing of contralateral forelimb |
|  | 2 | Forelimb hanging |
| Resistance to Lateral Displacement | 0 | Normal symmetric resistance |
|  | 1 | Weakened resistance on paretic side |
|  | 2 | No resistance on paretic side |
| Wire Grasp Strength | 0 | Symmetric power |
|  | 1 | Asymmetric power |
| Grasping Reflex | 0 | Grasps stick when forepaw gently touched |
|  | 1 | No grasping |
| Spontaneous Activity | 0 | Normal or near normal exploratory and grooming behavior |
|  | 1 | Reduced locomotion and spontaneous limb movements |
|  | 2 | Responsive to stimuli only (tactile, auditory) |
|  | 3 | Immobile and unresponsive to stimuli and/or absent acoustic startle reflex |
| **Total** | **16** |  |

**Supplemental Table 3: Modified Neurological Severity Score**
